# Observations on the Normal Diet

**Published:** 1889-06

**Authors:** G. Munro Smith

**Affiliations:** Lecturer on Physiology at the Bristol Medical School


THE BRISTOL

### Medico-Chirurgical Journal.

JUNE, 1889.

### OBSERVATIONS ON THE NORMAL DIET.

BY

G. Munro Smith,

*Lecturer on Physiology at the Bristol Medical School.*

What is the normal diet? How much solid food is required daily by a healthy adult, and what should be the relative proportions of the organic constituents? The tables compiled by physiologists on these important points contain striking discrepancies which can be easily accounted for; some of the statistics being taken from barracks, workhouses, and other institutions where great economy has to be practised, and consequently where farinaceous and fatty articles of food are chiefly used; whilst others are compiled from experiments on students or on the physiologist himself. Climate, season, temperament, age, and the nature of the work done are also modifying influences, so that perfect coincidence is not to be expected. But in order to satisfy myself, I induced some students at the School and others to help me to ascertain how much food was consumed by the average

Englishman in the twenty-four hours; and, if possible, the amount necessary for health.

Before detailing the results I arrived at, I wish to say that I do not consider moderate over-eating necessarily injurious. There are very few, I believe, who do not habitually take more food than is really essential; but, although I have been irresistibly led to this conclusion, I do not intend to apply the moral either to my neighbour or myself.

During the day an ordinary labourer has to do work which corresponds, roughly, to four hundred and fifty foot-tons, irrespective of that which is done by the heart and other involuntary muscles. With brain-workers this quantity is difficult to fix; but the daily destructive metabolism, which is the great criterion of work done, does not vary much amongst different occupations. This waste must be replenished by food, and it is important for us to know, apart from mere instinct or appetite, the amount required to make the income and expenditure equal. "Instinct" prompts a man to eat until he is satisfied; but satisfaction often comes too soon or too late, generally the latter, and by long practice the maximum of distension steadily increases.

We cannot, however, compete with our ancestors; for there is abundant evidence that the scholars, wits, and statesmen of the eighteenth century both ate and drank to an extent that very few can now imitate with impunity.

The following is Swift's idea of a dinner for seven people, one of whom had "no appetite":—

"*First course:* Sirloin of beef, fish, shoulder of veal and tongue.

"*Second course:* Almond pudding, fritters, chickens, black-puddings and soup.

"*Third course:* Hot venison-pasty, hare, rabbit, pigeons, partridges, goose, and a ham." A goose being in those days considered "too much for one man, but not enough for two."

The influence of diet on the secretions is well known; but I may mention, as an instance of this, that the proportion of nitrogen to carbon excreted on what I consider a healthy mixture of food-stuffs is about *one* to *fifteen;* whereas in a diet rich in meat, such as many people indulge in, it becomes *one* to *seven,* the carnivora having the quotient at *four and a half.*

As examples of discrepancy in food statistics, it may be interesting to notice that whilst Ranke considers 15 to 16 ounces of chemically dry food a sufficient quantity for an average man on average work, and Dr. Dalton gives 19½ ounces, Huxley gives 22½ ounces, Pye-Smith 20 to 32, and Dr. Parkes 23 ounces.

Again, whilst Ranke gives the proportion of nitrogenous to non-nitrogenous food as *one* to *three and a half,* Moleschott fixes it at *one* to *seventeen and a third.*

My method of experimenting was, briefly, this: The food at each meal was weighed as carefully as circumstances would permit, and the water was subtracted. Taking one food with another, the amount of water is rather over 50 per cent., but with vegetarians it is more than this.

This, however, is not always the case, some vegetarians eating quantities of legumes and of rice, both of which contain much less water than lean meat. Ordinary bread when a day old only contains about 40 per cent. of water, and I found that bread and cheese, mixed in the normal proportions for eating, lost only 37·5 per cent. by drying.

Refined experimenting was not possible, for this would mean modifying the diet, the very thing I wished to avoid. I generally added on a little to the results obtained, to make up for conscious or unconscious deductions from the daily routine which I fancy were sometimes made.

The conclusions I came to may be summed up thus:

1. Very many men eat considerably more than the most liberal tables, such as those of Parkes. It is not uncommon for an average-sized man, on very moderate work, to eat 25 or 27 ounces of chemically dry food a day.*

2. Amongst the working class of medical students the daily quantity was found to be about 19 ounces: the lowest, on *mixed* diet, was 15.6 oz.; but in this case the height and weight were below the average.

3. Women eat much less than men, after making allowances for differences in weight and work. Where a man eats 19 ounces, a woman of the same weight and of active habits eats only 14 or 15 ounces.

4. On a diet from which all meat is excluded, I have found from reliable experiments that 12 to 13 ounces per diem can comfortably feed a hard-working man.

Thus the average diet in one case (the experiments lasting one week) consisted of $12\frac{1}{3}$ *ounces* per diem. A lady aged 76, a strict vegetarian, who enjoys unusual vigour of mind and body, has for some years lived on a daily diet of under 10 ounces. It should be remembered that peas, beans, rice, and eggs, which are almost always taken by "vegetarians," are highly concentrated foods.

5. The arrangement of meals appears to make a considerable difference in the amount eaten, late diners usually consuming more than those whose chief meal is in the middle of the day. For example, a medical man whose average was $19\frac{1}{2}$ ounces per diem when dining at two o'clock, ate, as he calculated, 21 to 23 ounces daily when dining at seven. Most men who dine early have the same appetite for dinner at half-past seven or eight as if they had merely a light lunch previously.

6. A moderate amount of stimulants (especially in the form of wine) apparently increases the average: moder-

* When I give quantities I shall always mean chemically dry food.

ately free drinking diminishes it. I cannot, however, state this with anything like certainty.

7. The relative amount of nitrogenous and non-nitrogenous food can be varied within wide limits with *apparent* impunity, one man eating in the proportion of one to two, another in the ratio of one to fifteen or sixteen. This modifies the excretion of urea very considerably, however, and the organs concerned in the formation of the nitrogenous metabolites do much more work in the former class. Experience shows that these organs frequently suffer, probably from the over-strain put upon them, and it is important to realise this.

A diet consisting of one part nitrogenous to seven or eight non-nitrogenous is a good combination. This is greatly exceeded on the nitrogenous side by the majority of men and women, especially by the former.

8. A diet of 12 to 14 ounces of chemically dry food, digestible, and with the ingredients in proper proportion, is sufficient, I am convinced, to keep an averaged-sized man, on moderate work, in good health. As far as I can ascertain, however, the majority of people in England eat literally *twice* as much, and this, too, in a nation that has had bequeathed to it by intemperate ancestors a heritage of gout and allied diseases.

